# Multiple Forms of Sexting and Associations with Psychosocial Health in Early Adolescents

**DOI:** 10.3390/ijerph18052760

**Published:** 2021-03-09

**Authors:** Yu Lu, Elizabeth Baumler, Jeff R. Temple

**Affiliations:** 1 Department of Health and Exercise Science, University of Oklahoma, Norman, OK 73019, USA; 2 Center for Violence Prevention, Department of Obstetrics and Gynecology, University of Texas Medical Branch, Galveston, TX 77555, USA; elbaumle@utmb.edu (E.B.); jetemple@utmb.edu (J.R.T.)

**Keywords:** early adolescents, sexting, psychosocial health

## Abstract

Despite the recent surge of sexting research, the link between sexting and psychosocial health remains inconclusive. To address this gap in the literature, we examined the link between multiple forms of sexting and a range of psychosocial health problems. Data were from a randomized controlled trial of a school-based dating violence prevention program. Participants were 2199 early adolescents (49.8% female) aged 14 years and under (mean age = 13.53, SD = 0.50) enrolled in middle-schools in southeast Texas. Participants self-reported to be 35.4% Hispanic, 7.9% Non-Hispanic White, 26.2% Non-Hispanic Black, 18.6% Asian, and 11.9% other. Multilevel multivariate regressions found that pressured sexting was associated with hostility and aggressive temperament. Receiving unsolicited sexts was associated with depression, impulsivity, hostility, emotion dysregulation, and aggressive temperament. Forwarding sexts without permission was linked to hostility. Asking someone for sexts was linked to impulsivity and aggressive temperament, while being asked to send a sext was associated with depression, anxiety, impulsivity, hostility, emotion dysregulation, and aggressive temperament. Finally, consensual sexting was not significantly associated with poor psychosocial health of any type. Interventions should focus on preventing pressured sexting and teaching early adolescents on how to respond to being pressured to sext.

## 1. Introduction

Sexting, defined as the sending, receiving, or forwarding of sexually explicit messages, images, or videos through electronic means, has become an increasingly prevalent behavior among adolescents [1]. According to a recent meta-analysis [2], 14.8% of adolescents aged between 12 to 17 years have sent a sext, 27.4% have received a sext, and 12.0% have forwarded a sext without consent.

Despite the recent surge of sexting research, the link between sexting and psychosocial health is still inconclusive [1,3]. The majority of the existing research has focused on depression and anxiety, with mixed findings emerging. Several studies identified positive associations between sexting and depression [4,5,6,7,8,9,10], while others found no significant relationship [11,12,13]. Similarly, for anxiety, while a number of studies identified positive associations [5,8,14,15], others reported no significant relationship [11,12,13]. Beyond these two variables, sexting has been linked to higher impulsivity [13], greater psychological distress [4,16], higher levels of stress and lower self-esteem [8], conduct disorder [15], and borderline personality disorder features [17]. Contrarily, a study of Italian adolescents revealed no differences in psychological distress between those who did not sext, moderately sexted, and frequently sexted [18].

The equivocal findings regarding sexting and psychosocial health are likely due to diverse definitions and measures of sexting [19], as well as the failure to distinguish different forms of sexting (e.g., wanted vs. unwanted sexts) [1]. For example, Klettke et al. [8] examined four forms of sexting and found that sending sexts under coercion and receiving unwanted sexts were associated with higher levels of depression, anxiety, stress symptoms, and lower self-esteem, while no relationship was identified for sending sexts or receiving sexts. Another study found that anxiety was inversely associated with sending and receiving sexts, depression was positively associated with receiving sexts but not with sending sexts, while conduct disorder was positively linked to sending and receiving sexts [15]. These findings suggest that the type, form, or context of sexting is especially important in considering whether and how sexting is related to psychosocial health.

As Englander [20] noted, pressured, coerced, and other nonconsensual sexting is substantially more likely to be associated with negative outcomes, relative to consensual sexting. Indeed, we previously argued that consensual sexting between intimate partners may be developmentally common, and thus is not expected to be associated with poor psychological health [1]; however, when coerced or pressured, sexting can be linked to feelings of guilt, shame, and embarrassment. Thus, to disentangle the complex relationships between sexting and psychosocial health, research needs to differentiate various forms of sexting. However, to date, the majority of existing studies have focused on one or two types of sexting (e.g., sending a sext [13]; receiving a sext [16]; sending and receiving [15]) or a composite measure of various forms of sexting (e.g., sending, receiving, forwarding of nude photos/videos and sexually suggestive messages [14]; combination of sending and receiving sexts [6]). Further, only a limited number of psychosocial health problems (e.g., depression, anxiety) have been considered.

Attempts have been made to summarize existing research to draw a conclusion on the link between sexting and psychosocial health by conducting literature reviews [3] and meta-analyses [21]. However, these efforts are inherently limited by the state of the current literature. For example, a recent meta-analysis [21] identified positive associations between sending sexts and internalizing problems, providing some evidence for the link between sexting and psychosocial health. However, given the novelty of the research area, these analyses were based on only seven studies published in 2019, and were limited to sending sexts and to only two forms of psychosocial health (anxiety and depression combined). Thus, how other forms of sexting (e.g., receiving sexts, coerced sexts) relate to multiple forms of psychosocial health remains unclear. There is a debate in the literature on whether sexting is a modern expression of normative sexual development or a risk behavior that clusters with problematic behaviors like risky sexual behaviors, substance abuse, and violence [19,22,23,24]. Research is inconclusive on the link between sexting and psychosocial health, likely owing to the fact that most studies have not distinguished between consensual and nonconsensual forms of sexting.

To address these gaps in the literature, we explored multiple forms of sexting, including sending sexts (under pressure vs. without pressure/consensual), receiving unsolicited sexts, forwarding sexts without permission, asking someone for sexts, and being asked to send sexts, and examined their associations with a range of psychosocial health problems (i.e., symptoms of depression, anxiety, impulsivity, hostility, emotion dysregulation, and aggressive temperament). In response to a call for increased sexting research on younger adolescents [19], we examined these behaviors on middle-school-aged youth.

## 2. Materials and Methods

### 2.1. Participants

We used follow-up data from a randomized controlled trial of a school-based dating violence prevention program. Participants were 2199 early adolescents aged 14 years and under (mean age = 13.53, SD = 0.50, age ranged “12 or under” to “14” years) enrolled in the 7th (95.9%), 8th (0.7%), and 9th (3.2%) grade in 24 schools in southeast Texas, including 1095 (49.8%) females, 1091 (49.6%) males, and 13 (0.6%) of unknown gender. Participants were self-reported to be 35.4% Hispanic, 7.9% Non-Hispanic White, 26.2% Non-Hispanic Black, 18.6% Asian, 5.6% other, and 6.3% unknown/unreported.

### 2.2. Procedure

Participants were recruited during school hours through mandated classes (e.g., health, science, physical education) in 2019. Participants were informed of the follow-up surveys. Those who returned signed parental consent forms and gave assent completed paper-and-pencil surveys in class. During the follow-up years, students who were hard to track or were located outside of the original school completed the survey online. A total of 3738 students were recruited, and 3028 completed the baseline survey (Response Rate: 81.0%). In 2019, 2367 participants completed the first follow-up survey (retention rate: 82.6% of 2865 eligible participants). In this study, only those who were aged 14 years or younger were included in the analysis, resulting in a final sample of 2199. Participants received $5 gift cards for participating at baseline, and $10 at the follow-up survey. All surveys were private and confidential. The study was approved by the last author’s Institutional Review Board.

### 2.3. Measures

Sexting. Participants were asked to report yes/no to whether they had engaged in various forms of sexting, including *sent sexts under pressure* (“Have you ever sent a naked picture of yourself to another person after being pressured to send one through text, email, or things like Snapchat?”), *sent consensual sexts* (“Have you ever sent a naked picture of yourself to another person without being pressured to send one through text, email, or things like Snapchat?”), *received unsolicited sexts* (“Has anyone ever sent you a nude photo (not of you) through text, email, or things like Snapchat without you asking?”), *forwarded sexts without permission* (“Have you ever forwarded the nude photo to others without their permission?”), *asked someone for sexts* (“Have you asked someone to send naked pictures of them to you?”), and *been asked to send sexts* (“Have you been asked to send naked pictures of yourself through text, email, or things like Snapchat?”).

Depressive symptoms. The Center for Epidemiologic Studies Short Depression Scale [25] was used to measure depressive symptoms. Participants reported on a scale of 1 = rarely or never (less than 1 day) to 4 = more or all of the time (5–7 days) on how often they experienced 10 depressive symptoms (e.g., “I was bothered by things that usually don’t bother me”) during the past week. The scale had a Cronbach’s α of 0.75.

Anxiety. The Generalized Anxiety Disorder subscale of the Screen for Child Anxiety-Related Emotional Disorders [26] was used to measure anxiety. Participants rated on a scale of 1 (not true or hardly ever true) to 3 (very true or often true) on nine items, such as “I worry about how well I do things” in general situations. This scale had a Cronbach’s α of 0.88.

Impulsivity was measured with the four-item Impulsiveness Scale from the Teen Conflict Survey [27]. Participants responded on a scale of 1 (never) to 5 (always) to items such as, “I start things but have a hard time finishing them”. The scale had a Cronbach’s α of 0.79.

Hostility was measured with the Symptom Checklist-90 [28]. Participants provided their answers to the question, “In general, how often do you…?” on six items, such as, “…feel easily annoyed or irritated” on a scale from 1 (never) to 4 (most of the time). This scale had a Cronbach’s α of 0.87.

Emotion dysregulation was measured with 11 items from the Borderline Personality Features Scale for Children [29]. Participants answered on a scale of 1 (not at all true) to 5 (always true) to items such as, “My feelings are very strong. For instance, when I get mad, I get really mad. When I get happy, I get really happy”. The scale had a Cronbach’s α of 0.92.

Aggressive temperament was measured with the aggression subscale of the Early Adolescent Temperament scale [30]. Participants reported on a scale of 1 (almost always untrue) to 5 (almost always true) to six items, such as, “When I am angry, I throw or break things”. The scale had a Cronbach’s α of 0.85.

Demographic variables. Age, gender, and race/ethnicity were collected.

### 2.4. Data Analysis

Preliminary analyses were carried out in SPSS 27.0 for Mac [31] to examine variable means, standard deviations, frequencies, and Pearson correlations. The intraclass correlation (ICC) in this study ranged from 0.006 to 0.036. To account for school-level ICC, a multilevel multivariate regression analysis was performed in Mplus 8.5 [32] to examine the associations between sexting and psychosocial health, controlling for age, gender, race, and exposure to the intervention. The robust maximum likelihood estimation method was used. Missing data were treated with full information maximum likelihood [33].

## 3. Results

Among our early adolescent participants, 3.7% reported having sent a sext under pressure, 3.7% had sent a consensual sext, 20.5% had received an unsolicited sext, 2.6% had forwarded a sext without permission, 4.5% had asked someone for sexts, and 18.6% had been asked by someone to send sexts. As shown in Table 1, significant bivariate correlations were identified between all forms of sexting and all types of psychosocial health, except that anxiety was only correlated with sending sexts under pressure (*r* = 0.04, *p* < 0.05), receiving unsolicited sexts (*r* = 0.10, *p* < 0.01), asking someone for sexts (*r* = 0.04, *p* < 0.05), and having been asked to send sexts (*r* = 0.14, *p* < 0.01).

As shown in Table 2, multilevel multivariate regression analyses found that, after controlling for age, gender, race, intervention exposure, and other forms of sexting, sending sexts under pressure was positively associated with hostility (*ß* = 0.04, *p* < 0.05) and aggressive temperament (*ß* = 0.06, *p* < 0.05). Receiving unsolicited sexts was associated with depression (*ß* = 0.09, *p* < 0.001), impulsivity (*ß* = 0.10, *p* < 0.001), hostility (*ß* = 0.14, *p* < 0.001), emotion dysregulation (*ß* = 0.14, *p* < 0.001), and aggressive temperament (*ß* = 0.14, *p* < 0.001). Forwarding sexts without permission was associated with hostility (*ß* = 0.05, *p* < 0.05). Asking someone for sexts was associated with impulsivity (*ß* = 0.07, *p* < 0.05) and aggressive temperament (*ß* = 0.07, *p* < 0.05). Being asked to send sexts was associated with depression (*ß* = 0.12, *p* < 0.001), anxiety (*ß* = 0.09, *p* < 0.01), impulsivity (*ß* = 0.11, *p* < 0.001), hostility (*ß* = 0.11, *p* < 0.001), emotion dysregulation (*ß* = 0.11, *p* < 0.001), and aggressive temperament (*ß* = 0.08, *p* < 0.01). Contrarily, after controlling for age, gender, race, intervention exposure, and other forms of sexting, sending consensual sexts was not associated with poor psychosocial health of any kind.

## 4. Discussion

Using a large, ethnically diverse sample of early adolescents, we identified associations between multiple forms of sexting and psychosocial health. In distinguishing between consensual and pressured sexting, we found that poor psychosocial health was only associated with the latter. Importantly, bivariate correlations suggested that consensual sexting was significantly associated with all psychosocial health types, which may help explain the mixed findings in the extant sexting literature that rarely distinguishes between pressured and consensual sexting [3]. Indeed, the bivariate link in our study was likely accounted for by pressured sexting, as among the 81 participants who had sent a sext under pressure and 81 who had sent consensual sexts (123 total), 42 (30.8%) of the adolescents reported having sent both pressured and consensual sexts. This finding gives credence to the suggestion [20] that pressured sexting is the primary culprit in feelings of guilt, shame, and embarrassment [1], which may contribute to psychosocial health problems. Schools and other communities with limited resources may shift their focus to preventing pressured sexting and how to respond to being pressured to sext.

Consistent with prior research [8,16], receiving unsolicited sexts was also linked to psychosocial health. In a qualitative study [34] exploring how people define sexting, it was found that only consensual messages were viewed as sexting, whereas unwanted sexts were viewed as an assault, which helps explain the psychosocial consequences of unsolicited sexts. Contrary to a previous study that identified a positive link between forwarding sexts without permission and depression [6], we found that forwarding sexts without permission was “only” associated with hostility. This finding was not particularly surprising, given the aggressive nature of this behavior, and suggests that health promotion programs aimed at reducing anger may have the secondary benefit of preventing this form of electronic sexual assault.

Early adolescents who had been asked to send a sext were at a particularly high risk of reporting symptoms of depression, anxiety, impulsivity, hostility, emotion dysregulation, and aggressive temperament. It is possible that being asked to send sexts can serve as a precursor for pressured or coerced sexting, or that simply asking for sexts is tantamount to pressure, given the desire to “fit in” or to get someone to like you [35,36]. Notably, our findings suggest that sending sexts under pressure and being asked to send sexts were independently associated with psychosocial health. That is, they uniquely contribute to psychosocial health, indicating a potential additive effect between these forms of sexting and psychosocial health. In addition to targeting the youth who pressure others for sexts, interventions should address adolescents who have been asked to engage in sexting.

Several limitations should be noted. First, the study examined cross-sectional associations between sexting and psychosocial health, so no causal inferences can be made. Second, the number of participants who reported having sent sexts (pressured or consensual), asked for sexts, or forwarded sexts without consent was relatively small (ranged from 2.6–4.5% of the sample) in this sample of early adolescents, which could have contributed to non-significant findings in regression analyses. Despite the small sample size, significant associations with poor psychosocial health were still identified with several sexting forms (e.g., sending sexts under pressure, being asked to send sexts, receiving unsolicited sexts), making it particularly concerning and highlighting the need for intervention. Third, although the study examined a range of sexting forms and psychosocial health, the included variables were not exhaustive. Future research should consider examining other forms of sexting (e.g., coerced sexting, receiving solicited sexts) and relationship contexts of sexting (e.g., being asked to send sexts from romantic partners vs. peers). Fourth, the survey did not include mental health treatment histories, which may have contributed to the link between sexting and psychosocial health. Lastly, while out of the scope of the present study, future research should consider examining how the associations between sexting and psychosocial health may differ by gender, race/ethnicity, socioeconomic status, sexuality, and other demographic factors.

## 5. Conclusions

As one of the first studies to examine multiple forms of sexting and psychosocial health, we found that pressured sexting was associated with hostility and aggressive temperament, and that receiving unsolicited sexts and being asked to send sexts was associated with depression, impulsivity, hostility, emotion dysregulation, and aggressive temperament. Forwarding sexts without permission was linked to hostility. Contrarily, consensual sexting was unrelated to poor psychosocial health. These findings emphasize the importance of sexting education, with a particular focus on nonconsensual and pressured forms of sexting, as well as how to manage being asked to send sexts.

## Figures and Tables

**Table 1:** Variable frequencies, means, standard deviations, and bivariate correlations.

Variables	Frequency (%)/Mean (SD)	Scale Range	1	2	3	4	5	6	7	8	9	10	11
1. Sent sexts under pressure	81 (3.7%)	yes/no	-										
2. Sent consensual sexts	81 (3.7%)	yes/no	0.46 **	-									
3. Received unsolicited sexts	450 (20.5%)	yes/no	0.19 **	0.19 **	-								
4. Forwarded sexts without permission	58 (2.6%)	yes/no	0.34 **	0.27 **	0.17 **	-							
5. Asked someone for sexts	100 (4.5%)	yes/no	0.34 **	0.49 **	0.22 **	0.31 **	-						
6. Been asked to send sexts	407 (18.5%)	yes/no	0.32 **	0.35 **	0.49 **	0.24 **	0.31 **	-					
7. Depression	1.80 (0.52)	1–4	0.13 **	0.10 **	0.19 **	0.12 **	0.10 **	0.23 **	-				
8. Anxiety	1.84 (0.52)	1–3	0.04 *	0.04	0.10 **	0.03	0.04 *	0.14 **	0.49 **	-			
9. Impulsivity	2.24 (0.98)	1–4	0.10 **	0.07 **	0.18 **	0.10 **	0.12 **	0.20 **	0.50 **	0.36 **	-		
10. Hostility	1.80 (0.67)	1–5	0.13 **	0.09 **	0.24 **	0.12 **	0.11 **	0.23 **	0.47 **	0.31 **	0.54 **	-	
11. Emotion Dysregulation	1.98 (0.90)	1–5	0.15 **	0.14 **	0.25 **	0.11 **	0.11 **	0.26 **	0.67 **	0.53 **	0.55 **	0.49 **	-
12. Aggressive Temperament	1.60 (0.72)	1–5	0.18 **	0.17 **	0.24 **	0.14 **	0.17 **	0.23 **	0.39 **	0.22 **	0.46 **	0.62 **	0.50 **

Note. * *p* < 0.05, ** *p* < 0.01.

**Table 2:** Multilevel multivariate regression results.

Independent Variables	Depression	Anxiety	Impulsivity	Hostility	Emotion Dysregulation	Aggressive Temperament
	R^2^ = 0.09	R^2^ = 0.08	R^2^ = 0.06	R^2^ = 0.11	R^2^ = 0.14	R^2^ = 0.12
Sent sexts under pressure	0.051	0.002	0.011	0.044 *	0.041	0.061 *
Sent consensual sexts	−0.001	−0.006	−0.027	−0.034	0.033	0.041
Received unsolicited sexts	0.093 ***	0.028	0.100 ***	0.142 ***	0.141 ***	0.144 ***
Forwarded sexts without permission	0.055	0.016	0.039	0.051 *	0.030	0.036
Asked someone for sexts	0.024	0.041	0.067 *	0.038	0.039	0.065 *
Been asked to send sexts	0.118 ***	0.091 **	0.107 ***	0.110 ***	0.110 ***	0.080 **
Age	0.014	−0.018	0.027	0.023	0.014	0.032
Gender (male as reference)	0.134 ***	0.211 ***	0.043 *	0.102 ***	0.236 ***	0.061 *
Race/Ethnicity (White as reference)						
Hispanic	0.108 *	0.000	−0.109 **	0.059 *	0.040	0.024
Black	0.034	−0.055	−0.049	0.156 ***	−0.009	0.151 ***
Asian	0.069	0.111 **	−0.073 **	0.039	0.064	0.020
Other	0.089 **	0.047	−0.007	0.077 **	0.054	0.051 *

Note. The numbers are standardized coefficients. All results are adjusted for intervention exposure. * *p* < 0.05, ** *p* < 0.01, *** *p* < 0.001.

## References

[1] Temple J.R., Lu Y., Walrave M., Van Ouytsel J., Ponnet K., Temple J.R. (2018). Sexting from a Health Perspective: Sexting, Health, and Risky Sexual Behaviour. Sexting: Motives and Risk in Online Sexual Self-Presentation.

[2] Madigan S., Ly A., Rash C.L., Van Ouytsel J., Temple J.R. (2018). Prevalence of multiple forms of sexting behavior among youth: A systematic review and meta-analysis. JAMA Pediatr..

[3] Gassó A.M., Klettke B., Agustina J.R., Montiel I. (2019). Sexting, mental health, and victimization among adolescents: A literature review. Int. J. Environ. Res. Public Health.

[4] Dake J.A., Price J.H., Maziarz L., Ward B. (2012). Prevalence and correlates of sexting behavior in adolescents. Am. J. Sex. Educ..

[5] Dodaj A., Sesar K., Jerinić S.A. (2020). Prospective Study of High-School Adolescent Sexting Behavior and Psychological Distress. J. Psychol..

[6] Frankel A.S., Bass S.B., Patterson F., Dai T., Brown D. (2018). Sexting, risk behavior, and mental health in adolescents: An examination of 2015 Pennsylvania Youth Risk Behavior Survey data. J. Sch. Health.

[7] Gámez-Guadix M., De Santisteban P. (2018). “Sex Pics?”: Longitudinal predictors of sexting among adolescents. J. Adolesc. Health.

[8] Klettke B., Hallford D.J., Clancy E., Mellor D.J., Toumbourou J.W. (2019). Sexting and psychological distress: The role of unwanted and coerced sexts. Cyberpsychol. Behav. Soc. Netw..

[9] Van Ouytsel J., Van Gool E., Ponnet K., Walrave M. (2014). Brief report: The association between adolescents’ characteristics and engagement in sexting. J. Adolesc..

[10] Ybarra M.L., Mitchell K.J. (2014). “Sexting” and its relation to sexual activity and sexual risk behavior in a national survey of adolescents. J. Adolesc. Health.

[11] Burić J., Garcia J.R., Štulhofer A. (2020). Is sexting bad for adolescent girls’ psychological well-being? A longitudinal assessment in middle to late adolescence. New Media Soc..

[12] Gordon-Messer D., Bauermeister J.A., Grodzinski A., Zimmerman M. (2013). Sexting among young adults. J. Adolesc. Health.

[13] Temple J.R., Le V.D., van den Berg P., Ling Y., Paul J.A., Temple B.W. (2014). Brief report: Teen sexting and psychosocial health. J. Adolesc..

[14] Chaudhary P., Peskin M., Temple J.R., Addy R.C., Baumler E., Ross S. (2017). Sexting and mental health: A school-based longitudinal study among youth in Texas. J. Appl. Res. Child.

[15] Kim S., Martin-Storey A., Drossos A., Barbosa S., Georgiades K. (2020). Prevalence and correlates of sexting behaviors in a provincially representative sample of adolescents. Can. J. Psychiatry.

[16] Valiukas S., Pickering M., Hall T., Seneviratne N., Aitken A., John-Leader F., Pit S.W. (2019). Sexting and mental health among young Australians attending a musical festival: A cross sectional study. Cyberpsychol. Behav. Soc. Netw..

[17] Brinkley D.Y., Ackerman R.A., Ehrenreich S.E., Underwood M.K. (2017). Sending and receiving text messages with sexual content: Relations with early sexual activity and borderline personality features in late adolescence. Comput. Hum. Behav..

[18] Morelli M., Bianchi D., Baiocco R., Pezzuti L., Chirumbolo A. (2016). Sexting, psychological distress and dating violence among adolescents and young adults. Psicothema.

[19] Van Ouytsel J., Walrave M., Ponnet K. (2018). Adolescent sexting research: The challenges ahead. JAMA Pediatr..

[20] Englander E. (2019). What Do We Know About Sexting, and When Did We Know It?. J. Adolesc. Health.

[21] Mori C., Temple J.R., Browne D., Madigan S. (2019). Association of sexting with sexual behaviors and mental health among adolescents: A systematic review and meta-analysis. JAMA Pediatr..

[22] Barrense-Dias Y., Berchtold A., Surís J.C., Akre C. (2017). Sexting and the definition issue. J. Adolesc. Health.

[23] Rice E., Gibbs J., Winetrobe H., Rhoades H., Plant A., Montoya J., Kordic T. (2014). Sexting and sexual behavior among middle school students. Pediatrics.

[24] Temple J.R., Strasburger V.C., Zimmerman H., Madigan S. (2019). Sexting in youth: Cause for concern?. Lancet Child Adolesc. Health.

[25] Andresen E.M., Malmgren J.A., Carter W.B., Patrick D.L. (1994). Screening for depression in well older adults: Evaluation of a short form of the CES-D. Am. J. Prev. Med..

[26] Birmaher B., Khetarpal S., Brent D., Cully M., Balach L., Kaufman J., Neer S.M. (1997). The screen for child anxiety related emotional disorders (SCARED): Scale construction and psychometric characteristics. J. Am. Acad. Child. Adolesc. Psychiatry.

[27] Bosworth K., Espelage D. (1995). Teen Conflict Survey.

[28] Derogatis L.R., Rickels K., Rock A.F. (1976). The SCL-90 and the MMPI: A step in the validation of a new self-report scale. Br. J. Psychiatry.

[29] Crick N.R., Murray-Close D.I., Woods K. (2005). Borderline personality features in childhood: A short-term longitudinal study. Dev. Psychopathol..

[30] Capaldi D.M., Rothbart M.K. (1992). Development and validation of an early adolescent temperament measure. J. Early Adolesc..

[31] IBM Corporation (2020). IBM SPSS Statistics for Mac.

[32] Muthén L.K., Muthén B.O. (2017). Mplus User’s Guide.

[33] Graham J.W. (2012). Missing Data: Analysis and Design.

[34] Manning J. (2013). Interpretive theorizing in the seductive world of sexuality and interpersonal communication: Getting guerilla with studies of sexting and purity rings. Int. J. Commun..

[35] Van Ouytsel J., Van Gool E., Walrave M., Ponnet K., Peeters E. (2017). Sexting: Adolescents’ perceptions of the applications used for, motives for, and consequences of sexting. J. Youth Stud..

[36] Van Ouytsel J., Walrave M., De Marez L., Van Damme K., De Wolf R., Baccarne B., Vanhaelewyn B., Ponnet K. (2020). Teenage sexting on the rise? Results of a cohort study using a weighted-sample of adolescents. Sex. Health.

